# Subaortic Membrane Co-existing With Hypertrophic Cardiomyopathy: A Clinically Challenging Diagnosis

**DOI:** 10.7759/cureus.29580

**Published:** 2022-09-25

**Authors:** Mehr un Nisa Farhan, Hamza Akhtar, Hussein Al Sudani

**Affiliations:** 1 Internal Medicine, Shaikh Khalifa Bin Zayed Al-Nahyan Medical and Dental College, Lahore, PAK; 2 Cardiology, Einstein Medical Center Philadelphia, Philadelphia, USA; 3 Internal Medicine, Einstein Medical Center Philadelphia, Philadelphia, USA

**Keywords:** sam, lvot obstruction, echocardiography, hypertrophic cardiomyopathy, subaortic membrane

## Abstract

Subaortic Membrane is the most common type of subaortic stenosis. That, coexisting with hypertrophic obstructive cardiomyopathy (HOCM) is an extremely rare combination and clinically underappreciated. In this report, we will discuss an 18-year-old male patient who presented with chest pain and dyspnea due to fixed (sub-aortic membrane), as well as dynamic (HOCM), left ventricular outflow tract obstruction (LVOT) obstruction.

## Introduction

Hypertrophic cardiomyopathy (HCM) is defined as significant hypertrophy of the left ventricle with or without dynamic left ventricular outflow tract (LVOT) obstruction due to systolic anterior motion (SAM) of the mitral valve. Affecting one in 500 people, this disease is marked by phenotypic and genotypic heterogeneity and is the most prevalent, heritable cardiovascular disease [1].

Subaortic stenosis (SAS) is associated with the obstruction of the LVOT and is divided into two types, discrete membranous SAS and diffuse fibromuscular SAS [2]. Despite HCM and SAS being considered separate entities, rare cases of coexistence have been described, representing a diagnostic challenge for physicians [3]. HCM patients with SAS are at an increased risk of developing symptoms of heart failure, therefore proper diagnosis and identification of this rare entity are of utmost importance. Furthermore, preoperative identification of this morphologic abnormality allows for appropriate surgical planning [4] Echocardiography is the key technique to demonstrate the anatomical factors and the hemodynamic aspects accounting for LVOT obstruction [5].

## Case presentation

**Figure 1:**
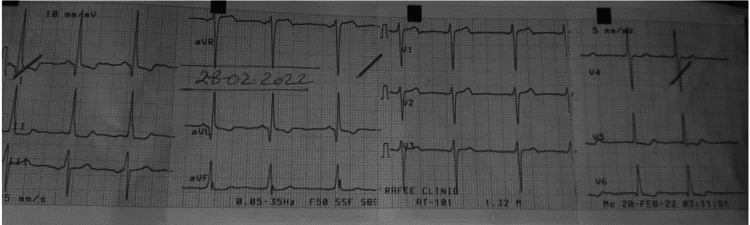
ECG of the patient shows sinus rhythm with T-wave Inversion in I, aVL, and V5-6.

An 18-year-old male presented to the ambulatory clinic with chief complaints of chest pain and shortness of breath with exertion. Symptoms started a few months before the presentation. There was no history of such symptoms in the family. On auscultation, a systolic ejection murmur of Grade III/VI intensity was heard along the left sternal border and the second intercostal space of the right sternal border. ECG shows sinus tachycardia at a rate of 112 bpm with ST-T changes in I, aVL, and V5-6 (Figure 1).

**Figure 2:**
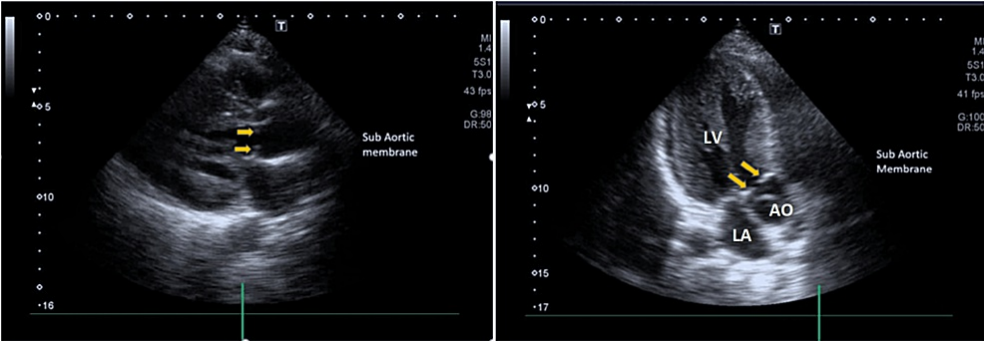
PLAX and APLAX view 2-D imaging showing severe LVH, septum is relatively thicker as compared to posterior wall (ASH). There is a sub-valvular membrane. Figure 2 (Left): 2-D PLAX in a diastolic frame (as the mitral valve is slightly open) showing severe LVH. The septum is relatively thicker as compared to posterior wall. Arrows pointing towards sub-aortic membrane. Figure 2 (Right): 2-D APLAX (three chamber) view in a systolic frame (as the mitral valve is closed and the aortic valve is slightly open). Arrows pointing towards echo dense sub-aortic valve membrane. PLAX=parasternal long axis; APLAX: apical long axis; 2-D: two-dimensional; LVH=left ventricular hypertrophy; ASH=asymmetric septal hypertrophy; LV=left ventricle; LA=left atrium; AO=aorta

**Figure 3:**
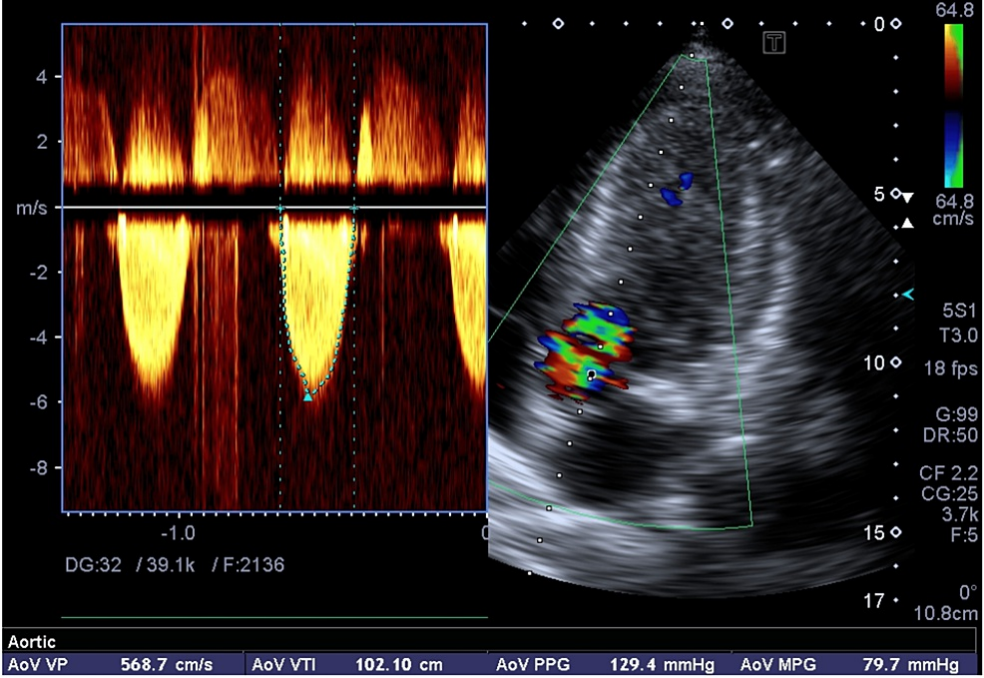
AP5CH view with color Doppler and spectral Doppler image. There is severe LVOT obstruction with peak velocity 5.68m/sec, peak gradient 129mmHg, and mean gradient 79mmHg.There is also spectral Doppler evidence of AR. AP5CH: apical five chamber; LVOT=left ventricular outflow tract; AR=aortic regurgitation

**Figure 4:**
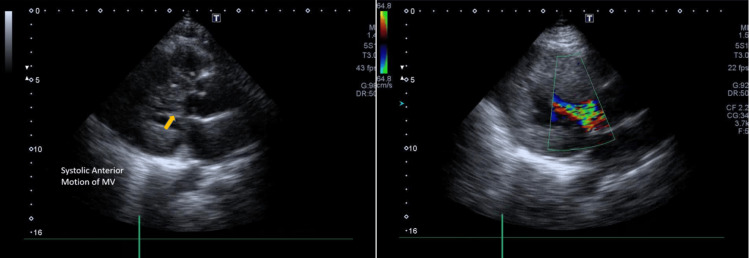
PLAX view showing systolic anterior motion of MV on 2-D image and turbulent flow in LVOT on color Doppler. To accurately identify the systolic anterior motion of the MV, an ECG is performed with the echo to time the events.  However, in this case, an ECG is not available. PLAX=parasternal long axis; MV=mitral valve; LVOT=left ventricular outflow tract

**Figure 5:**
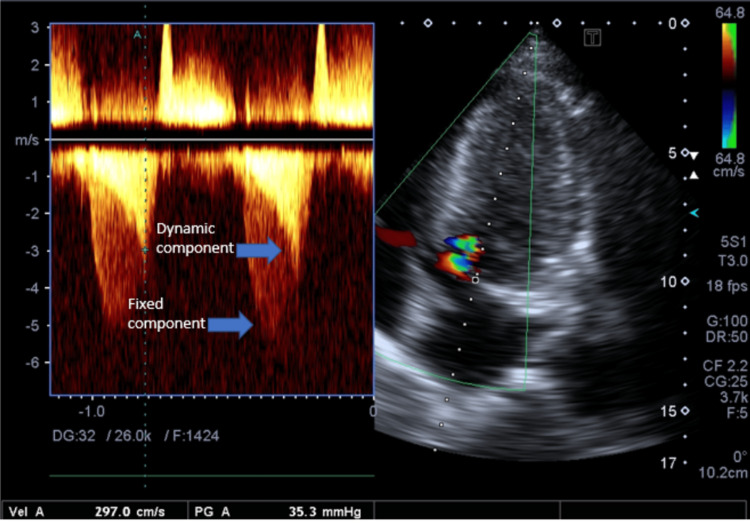
AP5CH view with color Doppler and spectral Doppler image There is double envelope of LVOT flow showing high flow velocities and gradients of dynamic and fixed components. Dynamic peak velocity 2.97m/sec and peak gradient 35mmHg. AP5CH: apical five chamber; LVOT=left ventricular outflow tract

Transthoracic echocardiography revealed severe left ventricular hypertrophy (Left ventricular interventricular septal thickness in diastole (LV IVSd) 18mm, Left ventricular posterior wall thickness in diastole (LV PWd) 17mm), normal LV regional wall motion, and no supportive evidence of any regional ischemia. LV systolic function was normal with LV EF 57% (Simpson’s Biplane). There was fixed LVOT obstruction (the presence of a subaortic membrane) (Figure 2)(Video 1 and Video 2) with a peak systolic gradient of 102mmHg and a mean gradient of 80mmHg (Figure 3). In addition, there was systolic anterior motion of the mitral valve (a diagnostic feature of HCM) (Figure 4) leading to dynamic LVOT obstruction with a peak systolic gradient of 35mmHg (Figure 5).

**Video 1:** PLAX view showing severe LVH. Septum is relatively thicker as compared to posterior wall (ASH). PLAX=parasternal long axis; LVH=left ventricular hypertrophy; ASH=asymmetric septal hypertrophy

**Video 2:** APLAX view showing SAM of the mitral valve and SAS. (Slow Motion Video) APLAX view (three chamber) showing mitral valve and aortic valve in systolic and diastolic frames. During systole, there is opening of aortic valve and there is echo dense membrane just beneath the valve. There is also SAM of mitral valve which is touching the interventricular septum during systolic phase. APLAX=apical long axis; SAM=systolic anterior motion; SAS=subaortic stenosis/subaortic membrane

The aortic valve was trileaflet with restricted opening due to sub-aortic membrane. Moderate aortic regurgitation was also present (pressure half-time (PHT) 263msec and diastolic flow reversal in descending aorta). Mild mitral regurgitation was also noticed. The patient was advised to get a transesophageal echocardiogram (TEE) and cardiac MRI followed by a surgical myomectomy. However, the patient voluntarily selected conservative treatment and was lost to follow-up.

## Discussion

HCM, defined as a wall thickness of >15mm in at least one left ventricular myocardial segment as determined by any imaging techniques, has a prevalence of at least one in 500 in the general population and is one of the most common genetic cardiac disorders with autosomal dominant inheritance [6,7]. The sub-aortic membrane is a rare congenital heart disease and is usually formed by a thin fibrous or occasionally muscular membrane of the LVOT [5]. Sub-aortic membrane associated with HCM remains a clinically challenging diagnosis in the adult population but has been reported to have a prevalence of three percent (15) in 466 patients with LVOT obstruction, out of which only 1.7% (8) had sub-aortic membrane and SAM-septal contact creating at least two discrete levels of outflow obstruction [4]. Often subaortic stenosis (SAS) can be misdiagnosed as HCM as both of them lead to left ventricular hypertrophy. However, both entities can be differentiated, as HCM causes asymmetric septal hypertrophy which is a primary pathological change and SAS causes left ventricular concentric hypertrophy which is a secondary pathological change. Moreover, SAM of the mitral valve is a diagnostic feature of HCM that is not seen in SAS.

Our patient’s echo exhibits a coexistent dynamic LVOT obstruction from HCM and a fixed obstruction from a subaortic membrane. Identification requires a high index of suspicion and raises important management and prognostic considerations. HCM patients with subaortic membranes may be at an increased risk of developing progressive heart failure symptoms [3]. It is critical to make the appropriate diagnosis as the treatment options are vastly different, as well as the implications of a diagnosis of HCM with regard to the risk of sudden death and family screening [5]. Echocardiology is the principal tool used for the diagnosis of SAS, which usually presents with the following characteristics: a slightly strong linear echo in the LVOT below the aorta or hypertrophic muscular structure protruding into the LVOT [2]. The optimal echo views for SAM of the mitral valve are the parasternal long axis (PLAX) and apical long axis (APLAX). For LVOT and aortic valve flow velocity and gradients, the optimal view is apical five chamber (AP5CH). Moreover, cardiac MRI and genetic testing can aid in the diagnosis of HCM. Combined with other diagnostic interventions, ECG can also help in the diagnosis of HCM. Common ECG features include precordial voltages and non-specific ST segment and T-wave abnormalities.

Our patient’s echo revealed severe asymmetric LVH with septal and anterior wall enlargement, SAM of the mitral valve, and the aortic valve having a restricted opening due to a sub-valvular membrane. An ECG should always be performed with the echo to time the events so as to accurately identify the SAM of the mitral valve. Identification of the underlying cause of heart failure is critical in patients with structural heart disease, as treatment strategies and prognosis differ based on etiology. HCM is usually treated with surgical septal resection or percutaneous alcohol ablation. However, percutaneous alcohol septal ablation would be ineffective in relieving obstruction due to sub-aortic membranes [4]. SAS is primarily treated by surgical removal of the sub-aortic membrane [2]. With respect to conservative treatments, simple HCM is mainly treated with beta-blockers [7], while patients with HCM combined with SAS, due to more severe LVOT obstruction, also need to use low-dose diuretics to reduce heart load and breathing difficulties [2]. 

## Conclusions

This case highlights a clinical scenario in which a patient has a subaortic membrane co-existing with the systolic anterior motion of the mitral valve and dynamic LVOT obstruction. It illustrates the key role of echocardiography in the assessment and diagnosis of such complex cases of cardiomyopathy.
